# A Thorough Navigation of miRNA's Blueprint in Crafting Cardiovascular Fate

**DOI:** 10.1002/hsr2.70136

**Published:** 2024-11-05

**Authors:** Abubakar Nazir, Olivier Uwishema, Sanobar Shariff, William Xochitun Gopar Franco, Noha El Masri, Nitsuh Dejene Ayele, Isabelle Munyangaju, Fatima Esam Alzain, Magda Wojtara

**Affiliations:** ^1^ Department of Medicine Oli Health Magazine Organization, Research and Education Kigali Rwanda; ^2^ Department of Medicine King Edward Medical University Pakistan; ^3^ Department of Medicine Yerevan State Medical University Yerevan Armenia; ^4^ Department of Medicine University of Guadalajara Guadalajara Mexico; ^5^ Faculty of Medicine Beirut Arab University Lebanon; ^6^ Department of Internal Medicine, Faculty of Medicine Wolkite University Wolkite Ethiopia; ^7^ Barcelona Institute for Global Health—Hospital Clínic Universitat de Barcelona; ^8^ Department of Medicine College of Medicine and General Surgery—Sudan University of Science and Technology; ^9^ Department of Medicine University of Michigan Medical School Ann Arbor Michigan USA

**Keywords:** cardiac diseases, gene expression, miRNA, vascular diseases

## Abstract

**Introduction:**

Cardiovascular diseases contribute significantly to global morbidity and mortality. MicroRNAs are crucial in the development and progression of these diseases by regulating gene expression in various cells and tissues. Their roles in conditions like atherosclerosis, heart failure, myocardial infarction, and arrhythmias have been widely researched.

**Materials and Methods:**

The present study provides an overview of existing evidence regarding miRNAs' role in cardiovascular disease pathogenesis. Furthermore, the study examines current state‐of‐the‐art technologies used in the study of miRNAs in cardiovascular disease. As a final point, we examine how miRNAs may serve as disease biomarkers, therapeutic targets, and prognostic indicators.

**Results:**

In cardiology, microRNAs, small noncoding RNA molecules, are crucial to the posttranscriptional regulation of genes. Their role in regulating cardiac cell differentiation and maturation is critical during the development of the heart. They maintain the cardiac function of an adult heart by contributing to its electrical and contractile activity. By binding to messenger RNA molecules, they inhibit protein translation or degrade mRNA. Several cardiovascular diseases are associated with dysregulation of miRNAs, including arrhythmias, hypertension, atherosclerosis, and heart failure. miRNAs can be used as biomarkers to diagnose and predict diseases as well as therapeutic targets. A variety of state‐of‐the‐art technologies have aided researchers in discovering, profiling, and analyzing miRNAs, including microarray analysis, next‐generation sequencing, and others.

**Conclusion:**

Developing new diagnostics and therapeutic approaches is becoming more feasible as researchers refine their understanding of miRNA function. Ultimately, this will reduce the burden of cardiovascular disease around the world.

## 1 Introduction

Despite identifying key primary interventions, cardiovascular disease (CVD) remains a leading cause of morbidity and mortality. Approximately 80% of sudden cardiac deaths globally result from coronary artery disease (CAD), while the remaining 20% are due to other cardiovascular conditions, including congenital heart disease, cardiomyopathies, left ventricular hypertrophy, and aortic valve disease [1, 2]. Risk factors for CVD include environmental factors, behavioral and lifestyle factors (like smoking), epidemiological factors (like pollution), and genetic factors [1, 2, 3].

The identification of genetic risk factors for CVD can be achieved using an analysis technique known as genetic association analysis, which starts to reveal novel genetic factors that contribute to the disease risk. Among the first genes implicated in CVD are those whose expression changes and increases the risk of myocardial infarction (MI) [4, 5]. MicroRNAs (miRNAs) are risk factors for CVD at specific loci. These small, double‐stranded RNA molecules are transcribed endogenously and regulate gene expression by blocking translation or promoting mRNA degradation [2]. In the heart, miR‐1 and miR‐133 are especially prevalent, with miR‐1 promoting cell proliferation and differentiation, while miR‐133 acts to inhibit these processes [6].

Developing therapeutic approaches requires an understanding of the genetic pathways underlying the development of atherosclerosis. Interventions may involve modifying the level of expression of a few significant genetic variables through various means, such as miRNAs. A significant portion of miRNAs are involved in the atherosclerotic process [7]. The correct creation of instruments for evaluating and managing the cardiovascular system requires the identification of these miRNAs [8].

Given that several miRNAs may be engaged in both reparative and degenerative processes in cardiomyocytes, numerous studies have emphasized the critical role that these miRNAs play in hypertrophy, cardiac failure, and CAD [9, 10]. Additionally, because the heart is a muscle by nature, various studies have concentrated on the role of so‐called myo‐miRNAs as regulators and indicators of cardiac injury.

Additionally, there have been a lot of therapeutic uses of miRNAs in the treatment of CVD in recent years. This is not surprising, given that new therapeutic uses are required to prevent CAD because the degeneration of cardiac cells in an ageing heart is not reversible due to the capture of cell division [11, 12]. To restore the mature myocardium's capacity for regeneration, efforts have been made recently to employ miRNAs to regulate the cell cycle [13].

The role of miRNAs in CVD has recently been studied using several state‐of‐the‐art technologies. As a result of these technologies, we now know much more about how miRNAs function, how they are regulated, and how they can be used as diagnostic or therapeutic tools in CVD.

Our aim is to provide an updated overview of miRNAs, technologies available for cutting‐edge diagnostic and therapeutic purposes, and their implications for CVD.

## 2 An Overview of miRNAs and Their Mechanisms of Action

miRNAs are short, noncoding RNA molecules, 19–25 nucleotides in length, that regulate gene expression. Their formation starts in the nucleus where RNA polymerase II transcribes them into primary miRNAs. These primary miRNAs are then capped, spliced, and polyadenylated. The Microprocessor complex, which includes DROSHA and DGCR8, processes the primary miRNA into precursor miRNA [14, 15, 16]. This precursor is transported to the cytoplasm by Exportin 5, where it is further processed by DICER and TRBP into a smaller RNA duplex. While some RNA molecules are degraded, others pair with AGO proteins to form the RNA‐induced silencing complex (RISC), which facilitates posttranscriptional gene silencing [14, 15, 16, 17, 18].

**Figure 1:**
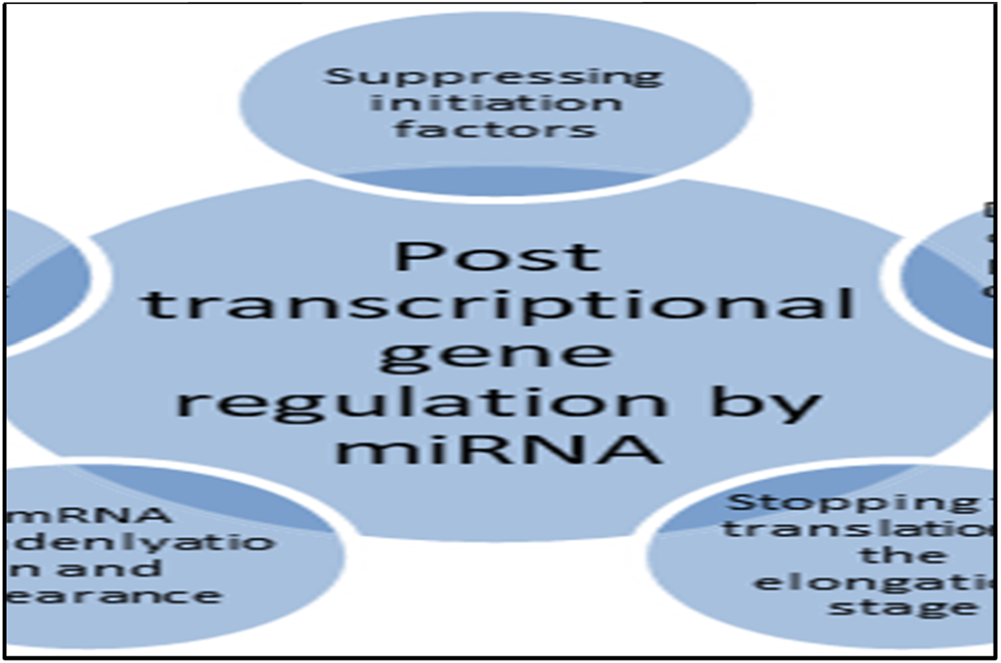
Different methods used by the miRNA to downregulate mRNA posttranscriptional steps.

Through targeting mRNA stability and translation, miRNAs play a significant role in posttranscriptional gene regulation (Figure 1). Genes can be upregulated or downregulated as a result of this process [19, 20]. A miRNA's downregulation process targets transcription in many ways: it suppresses essential initiating factors at the initiation or post‐initiation stage, promotes the degradation of a polypeptide chain forming during translation, processes the mRNA into cytoplasmic foci, and even facilitates its deadenylation and clearance [21, 22, 23]. Further, miRNAs can halt translation at the elongation stage, which results in slower protein synthesis [21, 22, 23]. On the other hand, miRNAs have the ability to upregulate genes [19]. Specific factors and cellular conditions must be present or absent for this upregulation to occur [19, 24]. In addition, this process has been shown to occur through competition with certain decay pathways, blocking repressive proteins, or even collaborating with some mRNP complexes [19, 24].

In this field of research, the application of in silico technologies could be regarded as fundamental [25]. The role of miR‐16 and hsa‐miR‐124 in the biomolecular processes that cause atherosclerosis was recently revealed in an article [26], indicating the possibility of using these biomarkers as therapeutic and diagnostic tools (theranoMiRNAs). A different experimental investigation showed the role of several miRNAs in CAD by combining an integrated strategy with an in silico analysis. These results were validated by the in silico evaluation conducted in a study by Sessa et al.,: miR‐133a modulates many processes related to the control of the frequency rate or contractions of the heart by actively acting on the TAGLN2 and FSCN1 genes [27]. These findings provide credence to the notion that this miRNA—which may be regarded as a theranoMiRNA—is solely associated with the modeling of heart functioning [27] (Figure 1).

MiRNA pathway plays an important role in recognizing and binding mRNA to its target. First, the miRNA recognizes the mRNA by recognizing a complementary seed sequence [28]. With the use of the 5' and even 3' regions of miRNA, this seed sequence can be found in several canonical and noncanonical ways [29, 30]. Furthermore, accessibility of the target plays a crucial role in the recognition of the target sequence by miRNAs [31]. Through special interactions with RNA binding proteins, RNA binding proteins further contribute to miRNA maturation. Genetic regulation and RNA processing are also influenced by these proteins [32, 33]. Similarly, miRNA and target complex stability facilitates the interaction between miRNA and target complexes. Thermodynamic reactions facilitate this stability [34].

## 3 miRNAs Implicated in CVD

### 3.1 Mirnas Associated With Atherosclerosis

In the context of CVDs, multiple miRNAs have been associated with a variety of cardiac and vascular events [35] (Table 1); among them is miR‐21 [36]. Neointimal hyperplasia of the smooth muscle wall of the arteries is greatly controlled by miR‐21 [37]. In terms of genetic regulation, miR‐21 is regulated by a long noncoding RNA known as (TUG1) [38]. Cellular production is enhanced and programmed cell death is promoted by this regulation [39]. Additionally, miR‐21 downregulates the tumor suppressor gene PTEN [38]. This suppression of PTEN initiates the phosphorylation and activation of the AKT kinase promoting cell growth and inhibiting death [38, 40]. Furthermore, miR‐21 deficiency has been associated with destruction of the ATP‐binding cassette transporter G1 (ABCG1) [40]. Damage to this transporter promotes and facilitates the formation of foam cells, resulting in atherosclerotic plaque formation and atherogenesis [41].

**Table 1:** Overview of the different miRNA mentioned with their different functions.

miRNA	Function
miR‐21	Hyperplasia of neointima Cellular production Cell death Foam cell formation
miR‐126	Angiogenesis Vascular stability Neointimal formation Neointimal proliferation Neointimal migration Cellular movement and survival Cytoskeletal remodeling Inhibiting leukocytes adherence to endothelium Antithrombotic role
miR‐155	Inflammatory processes Activates macrophages Facilitates the development of atherosclerosis

Atherosclerosis is also associated with miR‐126. Several studies have shown that this miRNA is abundant in highly vascularized organs and plays a significant role in preserving angiogenesis and vascular activity [42, 43]. The action of miR‐126 is also enhanced when hypoxic conditions exist [44]. In endothelium, miR‐126 regulates cellular movement and survival, cytoskeletal remodeling and vascular stability and multiplication [35, 36]. Furthermore, miR‐126 regulates Foxo3, antiapoptotic B‐cell lymphoma 2 (BCl2) and insulin receptor substrate 1 (IRS1) genes to control neointimal formation, proliferation, and migration [35, 37]. Additionally, this miRNA inhibits the vascular cell adhesion molecule 1 (VCAM1) [38], which limits leukocyte adhesion to endothelial cells. Lastly, it plays an important antithrombotic role [39]. Consequently, Tissue Factor, which activates the intrinsic coagulation system, is attenuated [39, 40]. Through its control of the PI3K/Akt antiapoptotic pathway and PTEN tumor suppressor gene expression, miR‐126 is capable of suppressing cellular death [41].

MicroRNA‐155 is linked to atherosclerosis development. This miRNA is modulated by long noncoding RNAs and protein‐coding mRNA [45]. By targeting different inflammatory cells, miR‐155 regulates inflammation in the body [46]. LDL, for example, promotes the expression of miR‐155 in macrophages [41]. By downregulating BL2, this miRNA polarizes macrophages into alternately activating M2 macrophages in atherosclerosis [45]. Atherosclerosis plaques are also formed due to BCL6 inhibition [46]. The development of atherosclerosis is largely due to this pathway [47] (Table 1).

#### 3.1.1 The Significance of miR‐1 for Heart Hypertrophy and Fibrosis

**Figure 2:**
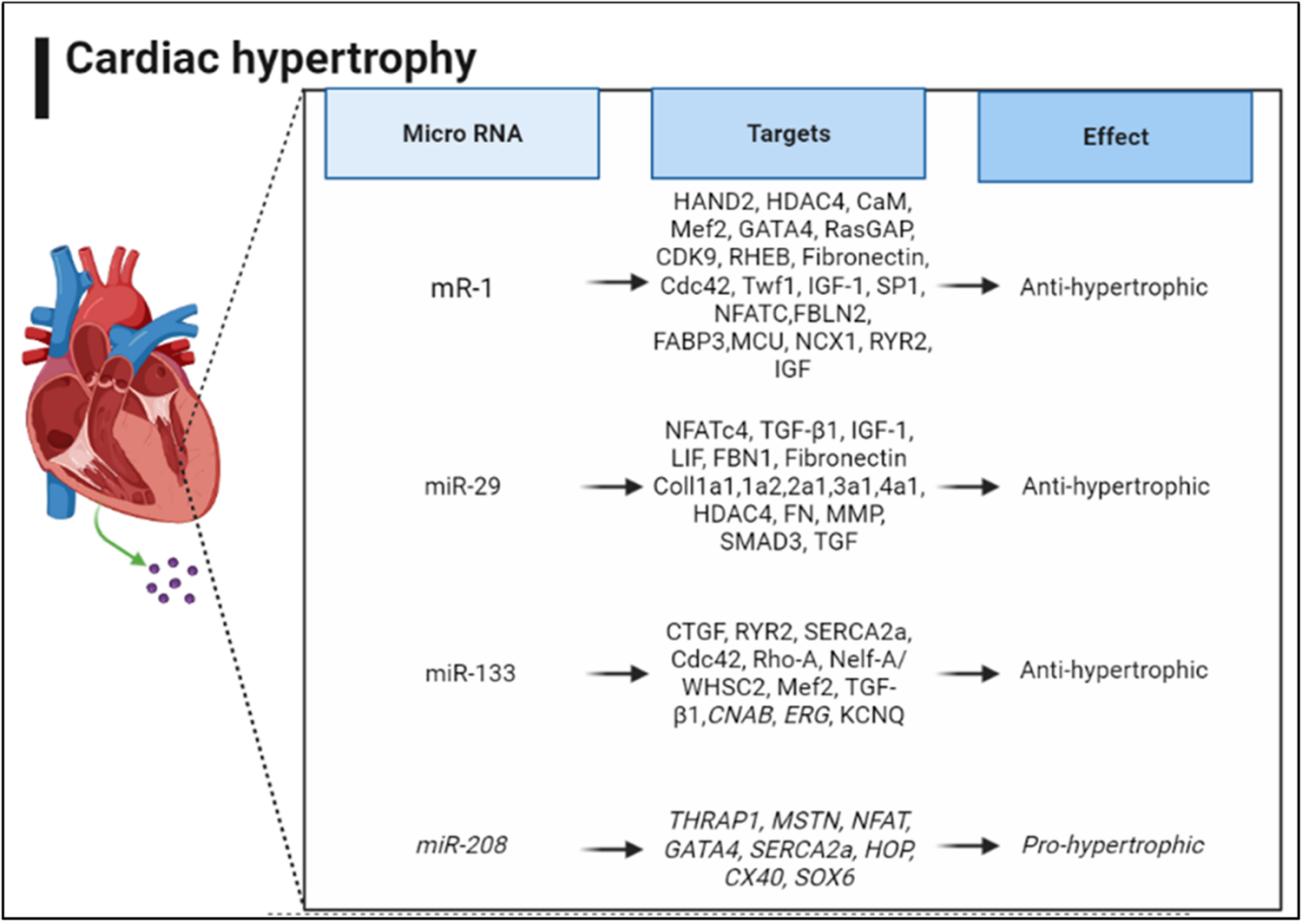
miRNAs involved in cardiac hypertrophy through cardiomyocyte size and fibrosis.

There are two miR‐1 molecules, miR‐1‐1 and miR‐1‐2, which are primarily expressed by cardiac precursor cells [48] (Figure 2). They regulate genes associated with cell differentiation and heart development [49, 50, 51]. These factors influence muscle differentiation [48, 49, 50, 51, 52, 53, 54, 55].

miR‐1 overexpression exerts anti‐hypertrophic effects while its downregulation promotes hypertrophy. It upregulates the mitochondrial calcium uniporter (MCU) and thus, increases calcium uptake. It has been seen in animal models that delivery of miR‐1 improves contractility and kinetics [56, 57, 58, 59]. Findings in animal models have shown that miR‐1 downregulation induces hypertrophy. The pro‐hypertrophic calcineurin‐NFAT pathway contributes to its protective effects [60, 61]. The expression of miR‐1 has been investigated in vitro on mouse cardiac cells. The reduction of key calcium homeostasis mediators, including calmodulin, GATA4, Mef2, and calcium‐dependent kinase II (CaMKII)‐histone deacetylase (HDAC) pathway, has been attributed to miR‐1 [48, 62, 63]. Other targets in this regard are the Sodium/Calcium exchanger protein (NCX1) and ryanodine receptor 2 (RYR2) [53]. Similar suppression of RasGAP, RHEB, CDK9, and Fibronectin has been observed in the context of miR‐1 gain‐of‐function [54, 55]. Protection against cyclin‐dependent kinase 6 retinoblastoma (CDK6‐rb), a significant contributor to hypertrophy, is further evidence of the effects of this compound. IGF/IGF‐1 and twinfilin actin‐binding protein 1 (TWF1) are inhibited by miR‐1, which reduces cardiac hypertrophy and cardiac fibrosis. Moreover, miR‐1 suppresses HAND2, known to promote ventricle cardiomyocyte expansion [48, 56] (Figure 2).

Targets of miR‐1 are summarized in Figure 2.

#### 3.1.2 Influence of miR‐208 in Myocardial Remodeling

The family of miRNAs known as miR‐208 has been identified primarily in cardiomyocytes [56]. They exhibit close ties with the alpha‐cardiac muscle with which they share loci and are co‐expressed [57, 58]. miR‐208a predominates in the left atrium, while miR‐208b is associated with the left ventricle and skeletal muscles [59, 60].

MHC gene expression and cardiac hypertrophy are directly intertwined. The switch from isoform α‐MHC to β‐MHC is an established phenomenon under stress conditions which, if chronically sustained, leads to cardiac remodeling and ultimately, cardiac hypertrophy [61, 62]. Cardiomyocyte enlargement without added proliferation is characteristic of this maladaptive process intended to make up for an increase in workload [63, 64, 65]. This leads to an increase in myofibrillar volume, in addition to the accumulation of collagen and other ECM elements. miR‐208 is considered to be pro‐hypertrophic, influencing cell growth and differentiation of cardiac precursors. Calcium overload plays a key role in such hypertrophic effects [56, 66]. Numerous research in both animals and humans have evidenced its upregulation under cardiac hypertrophy conditions both in animal and human studies [61]. The thyroid hormone signaling pathway is one of the mechanisms that confers its function. It does so by inhibiting thyroid hormone receptor cofactor; THRAP1 in addition to myostatin, and upregulating β‐MHC [67]. The role of thyroid hormones on cardiac hypertrophy is given due to their regulation of both α‐ and β‐MHC, cardiac troponin, Sarco/endoplasmic reticulum Ca^2+‐^ATPase (SERCA2a), and voltage‐gated potassium channels [68].

miR‐208a is essential for both pathological and physiological conditions [27, 69]. It is known to trigger apoptosis alongside oxidative stress [25]. Upregulation of miR‐208 has been linked to thickening of the myocardium [68]. The role of this gene in the development of fibrosis has been demonstrated in knockout models [65]. The homeodomain‐only protein (HOP) as well as GATA4 and CX40 are known to be adversely regulated for cardiac remodeling [66]. Another mechanism is via inhibiting the gene encoding sex‐determining region Y box 6 (SOX6). Inhibition of miR‐208a is posed as a therapeutic target for hypertrophy after studies that evidenced prevention of pathological remodeling, improved cardiac function, and associated survival [67].

MiR‐208b regulates cardiac hypertrophy, as evidenced across studies on dilated cardiomyopathy, MI, and arrhythmia [70]. Its involvement in Ca^2+^ handling is clarified by the fact that its overexpression is negatively associated with levels of SERCA2a. A further target of its activity is L‐type calcium channels (LTCCs) [53]. Mechanistically, its inhibition prevents pathological remodeling [28]. An interplay between miR‐208b and the *GIRK4* has been shown, with the former suppressing the latter, thereby reducing the formation and subsequent propagation of cardiac excitation [71].

#### 3.1.3 miR‐29's Effect on Cardiac Fibrosis and Cell Survival

miR‐29a, miR‐29b (particularly miR‐29b‐1 and b‐2) and miR‐29c are part of the miR‐29 cluster [72]. Fibroblasts are responsible for its production in the heart, which increases with age [73]. miR‐29 reduces the expression of extracellular matrix genes as the ones encoding for fibrillin 1 (FBN1), elastin, matrix metalloproteinase (MMP), and collagens; Col1α1, Col1α2, and Col3α1. The inverse relation between miR‐29 and collagens can also be observed in physiological growth as in exercise training [74, 75]. Upregulation of miR‐29b has been shown to be cardioprotective, reducing fibrosis, and improving cardiac function by inactivation of SMAD family member 3 (SMAD3) [76]. This is consistent with evidence of its downregulation in the setting of dilated cardiomyopathy [77]. Investigations carried out in vivo and in vitro elucidated how it prompts fibrosis and scar tissue formation through the activation of the TGF‐β and enhanced ECM deposition [78, 79].

Furthermore, research conducted in vitro demonstrated that miR‐29 regulates Wnt signaling, targeting Glis2, Gsk3b, Hbp1, and Ctnnbip1, as well, NFAT [72]. A second effect of its activities is to induce apoptosis by targeting secreted protein acidic and cysteine rich (SPARC) and preventing mybl2 from promoting survival. Its overexpression was inversely correlated to AKT3, in turn promoting apoptosis [73]. miR‐29 was shown to promote cell apoptosis through the suppression of IGF. It was also proved to influence apoptosis‐regulating proteins, namely MCL1 and BCL2 while promoting BCL2‐associated X protein (BAX) [46].

#### 3.1.4 The Crosslink Between miR‐1, miR‐133, and miR‐328 Within the Arrhythmogenic Processes

The miRNAs, miR‐1 and miR‐133 are clustered and thus co‐expressed. miR‐133 is made up of miR‐133a (which is further split in 133‐a‐1 and 133‐a‐2) along with miR‐133b [54, 66]. They play a major role in cardiogenesis through the stimulation of stem cell differentiation into mesodermal cells early in embryo development and later into cardiomyocytes [72]. In addition, they influence cardiac conductance and automaticity [73]. Both have been established as pivotal for the pathophysiology of states such as arrhythmias, cardiomyocyte hypertrophy, and fibrosis [74]. Their expression has been tied to activation of AKT and suppression of FOXO3A which is promoted by the increased activity of IGF‐1 [75]. Studies in physiological settings found identical effects from the downregulation of miR‐1 and miR‐133 on arrhythmogenesis [76].

miR‐1 has been associated with atrial and ventricular arrhythmias, acute coronary syndrome (ACS), and MI [77]. Its role in arrhythmia has been attributed to impaired kinetics and/or membrane trafficking systems arising from joint‐enhanced Ca^2+^, together with disrupted phosphatase activity at the RYR2 complex and modified expression of potassium (K^+)^ channels. Protein phosphatase 2A (PP2A), NCX1, calmodulin, and sorcin (SRI) have all been found to be regulated by miR‐1 [53]. This results in delayed after‐depolarizations. Studies have evidenced the shortening of the refractory period after the upregulation of miR‐1. Its pro‐arrhythmic properties are further attributed to the targeting of the gap junction channel gene; GJA1 and IK1 channel gene; KCJN2 [78]. It is a key component of cardiac remodeling by downregulating regarded genes such as the RHEB and RasGAP [79]. This function relates to its relationship with IGF‐1, with which it exhibits reciprocal negative feedback [80]. Interestingly, miR‐1 collaborates with miR‐133 to collectively target an array of genes encoding for ion channels and gap junctions, specifically HCN2, HCN4, B56alfa, calcium voltage‐gated channel subunit alpha1 C (CACNA1C*)*, and the iroquois homeobox protein 5 (IRX5) [81].

miR‐133, highly expressed in cardiac tissue as well found in skeletal muscle, has been reported to exert anti‐hypertrophic properties along with miR‐1. It is also reported to have a part in electrical repolarization, inhibiting adverse cardiac remodeling [82]. Supporting evidence from studies in vivo and in vitro show its downregulation under profibrotic conditions as TH‐mediated cardiac hypertrophy and dilated myocardiopathy [83]. It targets genes related to calcium homeostasis, cell growth and development, and extracellular matrix synthesis, such as Col1α1 and fibronectin [53, 84]. Its targets include genes involved in cell division regulation, calcium homeostasis, and heart hypertrophy; such as Cdc42, Rho‐A, Nelf‐A/WHSC2, RYR2, and SERCA2a [53, 55]. miR‐133 and the calcineurin downstream targets have been discovered to exhibit a mutually negative regulation [54]. It is also reported to suppress Snail‐I, Cyclin D2, connective tissue growth factor (CTGF), NFATc4, and guanosine diphosphate‐guanosine triphosphate (GDP‐GTP) have been found to be inhibited by it [77]. Mechanistically, miR‐133 overexpression regulates DNA methyltransferases, which helps to lessen cardiac fibrosis [63]. Another interesting mechanism by which it inhibits hypertrophy is by downregulating protein kinase C (PkC) and *G*_*q*_ protein, thus preventing Ca2^+^ influx [85]. miR‐133 was proven to target MMPs, which aids in degrading fibrotic tissue [2]. Other targets are Mef2 and the serum glucocorticoid responsive kinase‐1 (SGK1) and TGF‐β1 [86].

miR‐328, in contrast, promotes arrhythmogenic remodeling. Its overexpression has been demonstrated to foster cardiac hypertrophy and to be prevalent up to 3.5 times in patients with atrial fibrillation [77]. The mechanisms of this have been demonstrated during studies conducted in living organisms and in vitro proving the inhibition of SERCA2a, consequently increasing calcium uptake [56]. It is also established to target LTCC subunits encoding genes; CACNA1C, CCNB1, CACNB2, and CACNA2D, responsible for α1c/Cav1.2, β1, β2, and α2δ respectively. As a result, this shortens the duration of the action potential (APD) in the atrium while also decreasing the refractoriness, which promotes the onset of atrial fibrillation (AF) [87].

#### 3.1.5 Influence of miR‐21 and miR‐25 on Electrical Remodeling

**Figure 3:**
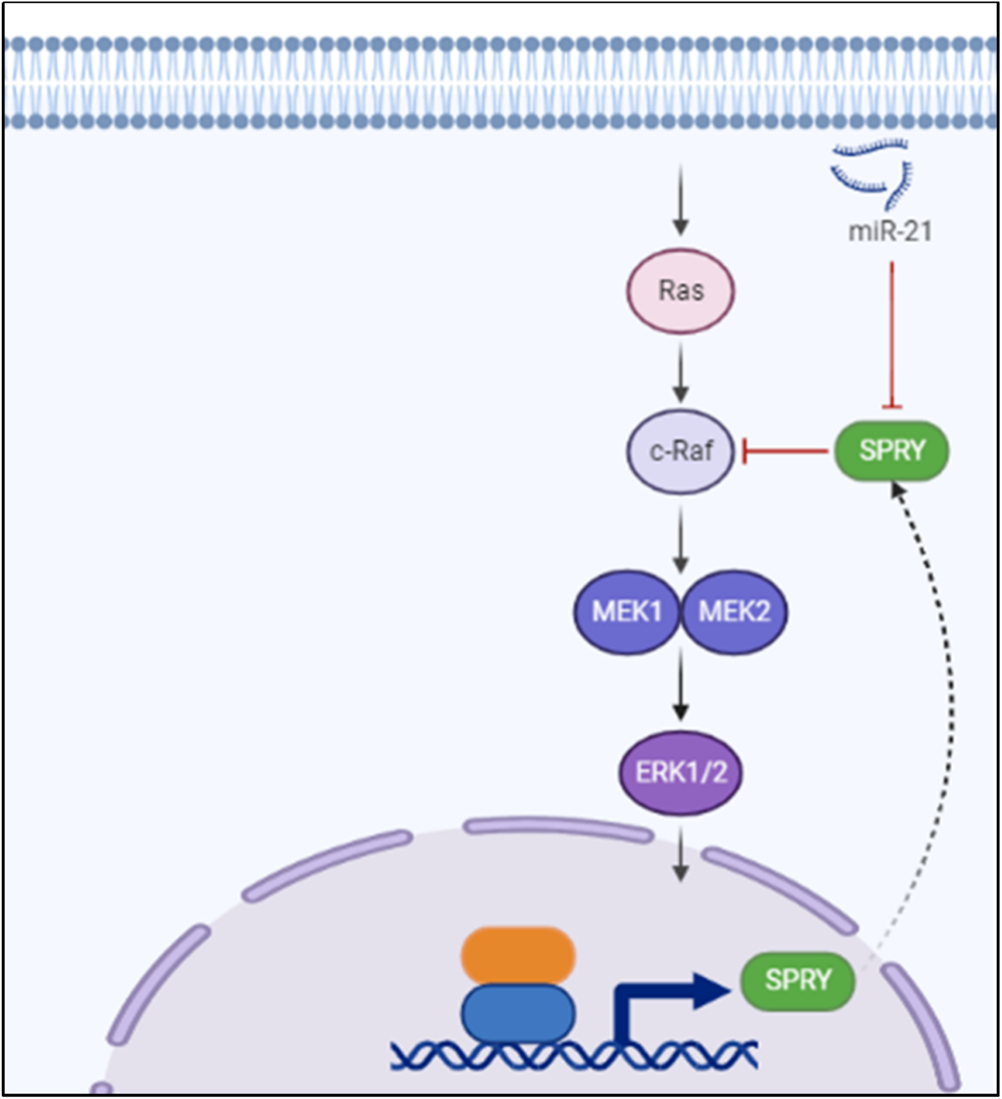
Effect of Sprouty on the Ras/Raf/ERK pathway. c‐RAF, cellular rapidly accelerated fibrosarcoma; ERK1/2, extracellular signal‐regulated kinase 1/2; MEK1, mitogen‐activated protein kinase 1; MEK2, mitogen‐activated protein kinase 2; RAS, rat sarcoma; SPRY, sprouty.

Electrical remodeling arises from changes in the expression of ion channels which reduce the APD and refractory period by disrupting calcium homeostasis [88] (Figure 3). This is often seen in arrhythmia. After hindering sprouty homologue 1 (Spry1), miR‐21, present in atrial myocytes and fibroblasts, influences cardiac structure and function by augmenting the ERK/MAPK activity, as depicted in Figure 3. miR‐21 plays an important role here, after the evidence on its role on inhibiting apoptosis, improving contractile function under hypertrophy, and promoting electrical remodeling [89]. Phosphate and tensin homolog (PTEN) levels and miR‐21 have been found to exhibit an inverse relationship, which in turn regulates PI3K expression for the electrical conduction and contractile function preservation downstream [90]. It decreases the density of LTCC currents by targeting genes involved in calcium homeostasis as LTCC [91]. Significantly, the levels of miR‐21 have been associated with cardiac remodeling as evidenced among patients afflicted with chronic atrial fibrillation. This makes it a useful MI biomarker [92] (Figure 3).

The role of miR‐25 is through calcium homeostasis with decreased SERCA2a and MCU expression [93]. Increased levels of potassium channel encoding gene, TASK‐1 along with Col1α2 extend the APD and lead to arrhythmia. Mechanistically, the expression of miR‐25 appears to be correlated to that of Col1α2 under AF. Similar findings were noted with miR‐21 [94].

#### 3.1.6 The Impact of miR‐499 Upon Expression of Ion Channels and Arrythmia Development

**Figure 4:**
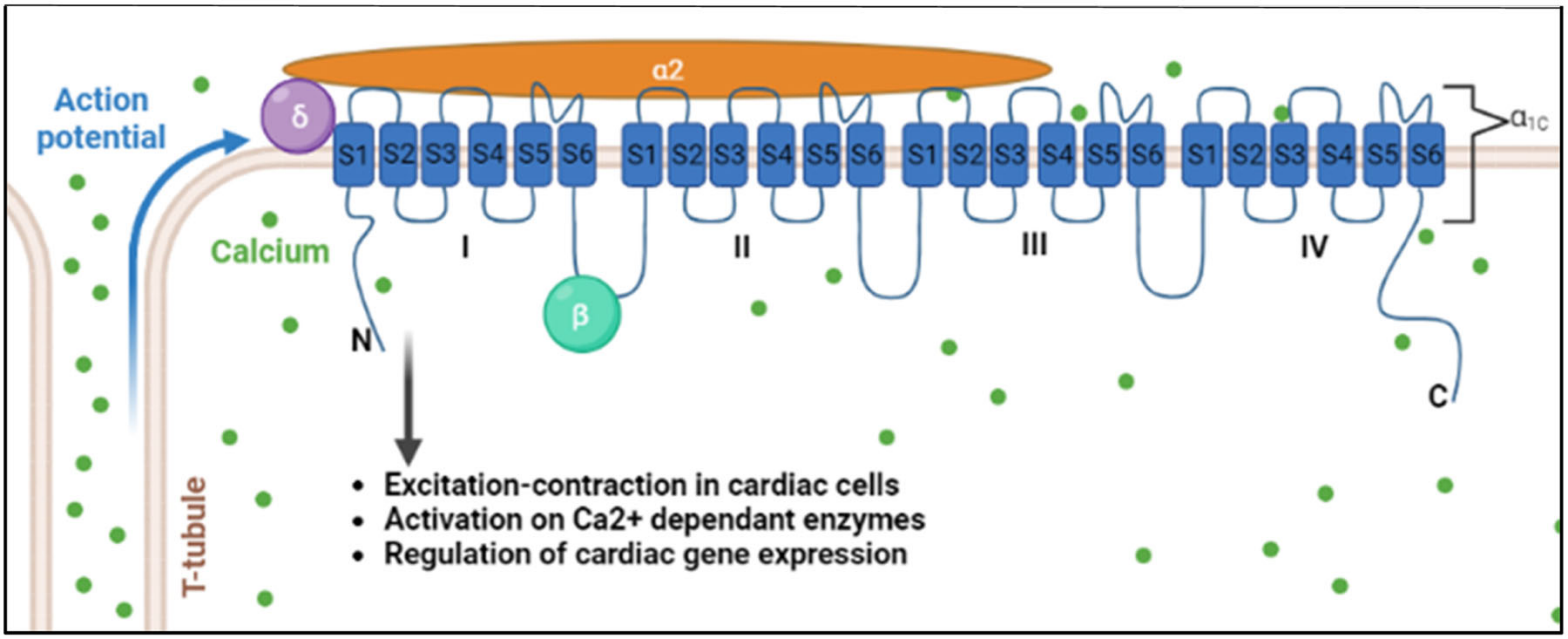
Conformation of the LTCC type CaV1.2 complex. The CaVα1C conforming the pore, comprises 4 homologous domains interlinked through transmembrane regions S6 to S1 in the next. N and C terminals are oriented at the cytoplasm. Auxiliary subunits CaVβ (purple) and CaVα2δ (orange) are also depicted.

The upregulation of miR‐499 has been linked to a rise of CVD (Figure 4). As well, it induces arrhythmia and has been shown to be downregulated halfway in atrial fibrillation [95]. Cardiomyocyte hypertrophy and myopathy are connected with higher levels of miR‐499, which is associated with arrhythmias and heart failure [96]. The significance of miR‐499 for the onset and progression of atrial fibrillation has been well established. miR‐499 is known to downregulate the potassium calcium‐activated channel encoding gene; KCNN3 in atrial fibrillation patients, thereby promoting electrical remodeling [97]. Experiments revealed that miR‐499 causes life‐threatening alterations in cardiomyocytes by modifying the expression of CACNA1C for the conformation of calcium channels in cardiomyocytes. LTCC type CaV1.2 is depicted in Figure 4 with conforming subunits (Figure 4).

### 3.2 Diagnostic and Prognostic Potential for miRNAs in CVD

#### 3.2.1 Use of miRNA as Biomarker of CVD Detection

The diagnostic and prognostic value of miRNA is being widely studied for patients with CVD, especially for heart failure and atherosclerotic heart disease patients (Table 2). For clinical use, a new biomarker should meet certain essential criteria, such as high sensitivity, availability for noninvasive testing, early disease detection capability, responsiveness to disease progression, accurate and reliable detection, long sample half‐life, affordability, and ease of interpretation for both physicians and patients [98]. miRNAs meet many of these criteria. For instance, miRNAs are produced by sequence‐specific amplification which makes them reliable markers; additionally, extracellular miRNAs enter the bloodstream packed by different carriers, which makes them resistant to harsh environmental conditions. The presence of miRNA in different body fluids will also guarantee less invasive test options. miRNAs exhibit diagnostic capabilities for CVDs that are equal to or surpass traditional biomarkers [99] (Table 2).

**Table 2:** Diagnostic potential of miRNAs for myocardial infarction and angina pectoris (AP).

MiRNA	Diagnostic potential	MiRNA alteration
miR‐499	It serves as a biomarker for acute myocardial infarction (AMI) and helps differentiate between STEMI and N‐STEMI AMI, with elevated levels seen in STEMI.	↑ ↑ STEMI
miR‐208a/b	Diagnostic or prognostic relevance for all cardiovascular diseases (CVD) Indicator for acute myocardial infarction (AMI) Differentiates between ST‐elevation myocardial infarction (STEMI) and non‐STEMI AMI Indicator for angina pectoris (AP) and distinguishes between AMI and AP	↑ ↑ STEMI
miR‐1	Biomarker for AMI and AP; differentiates between AMI and AP	↑
miR‐133a/b	Relevant for diagnosing or predicting all cardiovascular diseases (CVD). Serves as a biomarker for acute myocardial infarction (AMI) and angina pectoris (AP). Differentiates between AMI and AP. Distinguishes between STEMI and non‐STEMI AMI	↑ ↑ STEMI
miR‐145	Indicator of STEMI and severe AMI Differentiates between STEMI and non‐STEMI AM	↓ ↓ STEMI
miR‐451	Identification of plaque rupture Differentiation between STEMI and non‐STEMI AMI	↓ ↑ STEMI
miR‐155	Prognostic Impact: Risk of cardiac death following AMI Recognition: Identification of plaque rupture	↑
miR‐483‐5p	Identification of plaque ruptur	↑
miR‐19a	Predicts MI	↑
miR‐186	Early diagnosis of unstable angina	↑
miR‐21	Indicator for acute myocardial infarction (AMI)	↑
miR‐320a	Indicator for acute myocardial infarction (AMI)	↑
miR‐134	Differentiating between STEMI and non‐STEMI AMI	↑ STEMI
miR‐380	Prognostic significance for the risk of cardiac death following AMI	↑ in patients with an elevated risk of cardiac mortality
miR‐150	Prognostic significance for the risk of cardiac death following AMI	↑ ↓
miR‐106a‐5p	Forecasting future myocardial infarction	↑
miR‐132, miR‐140‐3p, miR‐210	Indicators of cardiovascular mortality	↑
miR‐126	Forecasting future myocardial infarction	↑

### 3.3 Prognostic Value of miRNA in Predicting CVD Outcomes

Besides their diagnostic applications, miRNAs are valuable for predicting CVD outcomes. Numerous studies emphasize their role in cardiac development and the pathological processes leading to heart diseases, including heart failure. For instance, miR‐1, miR‐133a, miR‐208, and miR‐499 are prominent in heart tissue, crucial for early heart development and cardiomyocyte differentiation. Abnormal levels of these miRNAs are associated with heart diseases such as heart failure and processes like arrhythmias, apoptosis, hypertrophy, fibrosis, and reverse remodeling. Consequently, miRNAs are anticipated to play a crucial role in predicting CVDs [100, 101].

#### 3.3.1 Challenges and Limitations in miRNA‐Based Diagnostics

Despite the numerous advantages of using miRNA, its application as a biomarker faces several limitations. The primary challenges include the high costs associated with testing and variations in miRNA expression that may not be exclusively linked to disease pathology. Differences in miRNA levels among populations could be attributed to ethnic, geographical, age‐related, or sex‐related factors, which need to be accounted for in the results. Other obstacles include the incomplete scientific understanding of miRNA functions, the complexity of biological processes they regulate, the vast number of miRNAs to study, and noncardiac conditions such as cancer, infections, or drug use that can influence miRNA expression. Additionally, the absence of standardized references to rule out confounding factors complicates their use. Therefore, before miRNAs can be reliably used as clinical biomarkers, extensive mechanistic studies in diverse, well‐characterized cohorts and various clinical settings are necessary [102, 103].

### 3.4 Therapeutic Applications of miRNAs in CVD

#### 3.4.1 Potential of miRNA‐Based Therapeutics for CVD Treatment

CVDs highlight the limitations of traditional pharmacotherapy, while miRNA‐based drugs have shown significant advancements in both preclinical and clinical trials [104] (Table 3). Unlike conventional drugs that target specific cellular pathways, miRNAs influence entire functional networks. Despite encouraging initial findings, no miRNA‐based treatment for CVDs has yet received approval [105, 106, 107, 108, 109, 110, 111, 112, 113, 114, 115, 116] (Table 3).

**Table 3:** Different miRNAs identified for treatment or CVS in animal studies.

MiRNA	Use in the treatment of	Ref
miR‐22	To prevent cardiac autophagy and post‐MI remodeling	[117]
miR‐99a	Enhanced left ventricular function and survival four weeks post‐myocardial infarction	[118]
miR‐320	Decreased myocardial fibrosis and apoptosis during left ventricular remodeling	[119]
anti‐miR‐15	Cardiomyocytes’ resistance to cell death caused by hypoxia‐induced damage	[120]
miR‐499 inhibitor	It improves symptoms of cardiac hypertrophy, heart failure, or myocardial infarction, or slows the progression from cardiac hypertrophy to heart failure.	[121]

Together, these studies indicate that miRNA‐based therapies utilizing modified oligonucleotides hold promise as therapeutic agents for patients experiencing a major acute MI, potentially influencing cardiac remodeling and preserving heart function following ischemic injury [105]. Although promising, the use of miRNA‐based therapeutics has certain limitations. Studies have shown that certain miRNAs have sex preference when used as therapeutic agents; in addition, external administration of miRNAs will not have a similar effect to that of the studied biological effect of miRNA.

#### 3.4.2 Strategies for Modulating Mirna Expression and Activity

There are several approaches to adjust miRNA expression and activity for potential disease treatment. A widely used method involves ex vivo alteration of intracellular miRNA levels by introducing miRNA mimics or anti‐miRNAs into cell cultures. MiRNA mimics are synthetic RNA duplexes that imitate the function of the desired miRNA, while anti‐miRNAs are chemically modified antisense oligonucleotides designed to bind and neutralize the target miRNA. This manipulation can either enhance (gain‐of‐function) or reduce (loss‐of‐function) the activity of the target miRNA. Several approaches are employed for the intracellular delivery of these compounds, including direct transfection of synthetic nucleotides and delivery via viral or plasmid vectors. However, challenges in clinical applications remain, particularly concerning the administration routes and drug concentration, which have yet to be resolved [106].

#### 3.4.3 Preclinical and Clinical Studies of miRNA‐Based Therapies

There is a variety of research on miRNA‐based treatments for CVDs, although most remain at the preclinical development stage. The impact of miRNAs on CVD has been less significant than expected, especially when compared to progress in other areas like miravirsen, RG‐101, cobomarsen, and AZD4076. Insights from both preclinical and clinical trials with inhibitory oligonucleotides, including those that were discontinued, are proving crucial for designing effective miRNA‐targeting therapies for cardiovascular conditions [107, 108, 109, 110, 111, 112, 113, 114, 115, 116] (Table 4).

**Table 4:** Major miRNA‐based therapeutics which are in the development phase for CVD [107].

Type of Molecule	Pathology	miRNA being targeted	Stage of development
MGN 1374	Post‐myocardial infarction treatment	miRNA 15 miRNA 195	Preclinical stage
MGN 4220	Cardiac fibrosis treatment	miR‐29	Preclinical Stage
MGN 9103	Chronic heart failure treatment	miR‐208	Preclinical Stage

### 3.5 Conclusion and Future Directions

Overall, miRNAs regulate gene expression and play an important role in numerous biological processes. Cellular homeostasis and overall physiological function depend heavily on their ability to regulate posttranscriptional gene expression.A better understanding of miRNAs' roles in CVD could provide insight into disease mechanisms and lead to new diagnostic and therapeutic approaches.

We have greatly enhanced our understanding of miRNA's role in CVDs through cutting‐edge technologies, and these technologies are crucial to developing diagnostic tools, therapeutic strategies, and personalized medicine approaches.

## Author Contributions

**Abubakar Nazir:** Visualization, writing–original draft, writing–review and editing. **Olivier Uwishema:** writing–original draft, writing–review and editing. **Sanobar Shariff:** supervision, visualization, writing–original draft, writing–review and editing. **William Xochitun Gopar Franco:** writing–original draft, writing–review and editing. **Noha El Masri:** writing–original draft, writing–review and editing. **Nitsuh Dejene Ayele:** writing–original draft, writing–review and editing. **Isabelle Munyangaju:** writing–original draft, writing–review and editing. **Fatima Esam Alzain:** writing–original draft, writing–review and editing. **Magda Wojtara:** writing–original draft, writing–review and editing.

## Ethics Statement

The authors have nothing to report.

## Conflicts of Interest

The authors declare no conflicts of interest.

## Transparency Statement

The lead author Abubakar Nazir affirms that this manuscript is an honest, accurate, and transparent account of the study being reported; that no important aspects of the study have been omitted; and that any discrepancies from the study as planned (and, if relevant, registered) have been explained.

## Data Availability

The authors have nothing to report.
